# High-Definition Mapping of Retroviral Integration Sites Defines the Fate of Allogeneic T Cells After Donor Lymphocyte Infusion

**DOI:** 10.1371/journal.pone.0015688

**Published:** 2010-12-22

**Authors:** Claudia Cattoglio, Giulietta Maruggi, Cynthia Bartholomae, Nirav Malani, Danilo Pellin, Fabienne Cocchiarella, Zulma Magnani, Fabio Ciceri, Alessandro Ambrosi, Christof von Kalle, Frederic D. Bushman, Chiara Bonini, Manfred Schmidt, Fulvio Mavilio, Alessandra Recchia

**Affiliations:** 1 IIT Unit of Molecular Neuroscience, Istituto Scientifico H. San Raffaele, Milan, Italy; 2 Center for Regenerative Medicine, University of Modena and Reggio Emilia, Modena, Italy; 3 Department of Translational Oncology, National Center for Tumor Diseases (NCT) and German Cancer Research Center (DKFZ), Heidelberg, Germany; 4 Department of Microbiology, University of Pennsylvania School of Medicine, Philadelphia, Pennsylvania, United States of America; 5 Center for Statistics in Biomedical Sciences, Università Vita-Salute San Raffaele, Milan, Italy; 6 Experimental Hematology Unit, PIBIC, Division of Regenerative Medicine, Gene Therapy and Stem Cells, Istituto Scientifico H. San Raffaele, Milan, Italy; 7 Hematology Unit, Istituto Scientifico H. San Raffaele, Milan, Italy; Institut Pasteur, France

## Abstract

The infusion of donor lymphocytes transduced with a retroviral vector expressing the HSV-TK suicide gene in patients undergoing hematopoietic stem cell transplantation for leukemia/lymphoma promotes immune reconstitution and prevents infections and graft-versus-host disease. Analysis of the clonal dynamics of genetically modified lymphocytes *in vivo* is of crucial importance to understand the potential genotoxic risk of this therapeutic approach. We used linear amplification-mediated PCR and pyrosequencing to build a genome-wide, high-definition map of retroviral integration sites in the genome of peripheral blood T cells from two different donors and used gene expression profiling and bioinformatics to associate integration clusters to transcriptional activity and to genetic and epigenetic features of the T cell genome. Comparison with matched random controls and with integrations obtained from CD34^+^ hematopoietic stem/progenitor cells showed that integration clusters occur within chromatin regions bearing epigenetic marks associated with active promoters and regulatory elements in a cell-specific fashion. Analysis of integration sites in T cells obtained *ex vivo* two months after infusion showed no evidence of integration-related clonal expansion or dominance, but rather loss of cells harboring integration events interfering with RNA post-transcriptional processing. The study shows that high-definition maps of retroviral integration sites are a powerful tool to analyze the fate of genetically modified T cells in patients and the biological consequences of retroviral transduction.

## Introduction

Peripheral blood donor lymphocytes promote immune reconstitution and anti-tumor activity in patients transplanted with allogeneic hematopoietic stem cells (HSCs) for the therapy of leukemia and lymphoma. The efficacy of donor lymphocyte infusion (DLI) is limited, however, by the risk of graft-versus-host disease (GvHD), a severe and often lethal complication. Expression of a suicide transgene - the herpes simplex virus thymidine kinase (HSV-TK) - in donor T cells allows an efficient control of GvHD by administration of the antiviral drug ganciclovir [1], [2], [3], [4]. DLI with TK-transduced T cells promotes immune reconstitution and graft-versus-leukemia (GvL) reaction, and prevents infectious complications and relapse, in patients undergoing both HLA-identical [1], [3] or HLA-haploidentical [4] HSC transplantation. In 45 patients treated in both contexts, GvHD was controlled in 100% of the cases, with no loss of antiviral and anti-tumor activity [3], [4]. In all cases, T cells were transduced with SFCMM, a vector derived from the Moloney murine leukemia retrovirus (MLV) expressing HSV-TK and a truncated version of the low-affinity nerve growth factor receptor (ΔLNGFR) as a marker for cell purification [1]. Neither retroviral integration nor the expression of HSV-TK and ΔLNGFR appeared to cause adverse effects in patients treated with transduced donor T cells [5], [6], [7].

The clinical use of MLV-derived vectors has raised significant safety concerns after the occurrence of lymphoproliferative disorders and pre-malignant clonal expansion in patients treated for X-linked severe combined immunodeficiency [8], [9] or chronic granulomatous disease [10], [11]. In all cases, the vector integrated in the proximity of proto-oncogenes and caused their deregulation. Similar integration events were observed in patients' cells in other clinical trials but did not cause adverse effects [12], [13], suggesting that other factors, such as cell context, vector design, patient's genetic background and conditioning regimens may contribute to neoplastic progression. Integration of MLV and MLV-derived vectors is non-random, with specific preferences for promoters and regulatory regions of active genes, although the molecular mechanisms underlying these preferences remain unknown [14]. We recently showed that MLV-derived vectors integrate preferentially in hot spots around cell-specific genes, enriched in defined subsets of transcription factor binding sites (TFBSs), and suggested that MLV pre-integration complexes (PICs) are tethered to transcriptionally active regulatory regions engaged by basal components of the RNA polymerase II (Pol II) transcriptional machinery [15], [16]. On this basis, integration patterns and frequently targeted loci are expected to be cell-specific, and should be determined for each cell type. As a consequence, the risk of causing insertional oncogenesis may vary when targeting different cell types (e.g., hematopoietic progenitors *vs*. T lymphocytes). Determining cell-specific integration patterns *in vitro* and tracking integration events *ex vivo* in treated patients may help in defining specific genotoxic risk in specific clinical contexts.

We used linear amplification-mediated PCR (LAM-PCR) and pyrosequencing to build a genome-wide, high-definition map of >8,000 integration sites of the SFCMM retroviral vector in the genome of peripheral blood T cells from two different donors. Gene expression profiling and bioinformatics were used to associate integration clusters to transcriptional activity and to genetic and epigenetic features of the T-cell genome. Comparison with matched random controls and with integrations obtained from CD34^+^ multipotent hematopoietic progenitor cells (HPCs) showed that MLV integrations cluster within chromatin regions bearing cell-specific epigenetic marks associated with active promoters and regulatory elements. Analysis of ∼1,000 integrations in T-cells obtained *ex vivo* two months after infusion in two patients treated with HLA-haploidentical HSC transplantation showed no evidence of clonal expansion but rather clonal loss of T cells harboring certain integration events.

## Results

### High-definition mapping of MLV integrations in pre- and post-infusion T cells

The study was carried out on two patients (TK38 and TK47) enrolled in a phase-II clinical trial aimed at proving the efficacy of the infusion of TK-transduced donor T cells in promoting immune reconstitution and preventing GvHD after haploidentical HSC transplantation for high-risk leukemia [4]. Peripheral blood T cells were obtained by leukapheresis from the donors, transduced with the SFCMM-3 retroviral vector expressing HSV-TK and ΔLNGFR, and immunoselected for LNGFR expression, as previously described [3]. TK cell infusion was followed by a rapid (20 and 13 days after infusion in TK38 and TK47, respectively) and sustained immune reconstitution, documented by increasing numbers of circulating T lymphocytes. Two months after infusion, transduced T cells (<10% of the total T cells in both patients) were retrieved from the peripheral blood mononuclear cell fraction and immunoselected for LNGFR expression. DNA was extracted from 10^7^ cells, and vector-genome junctions were amplified by linear amplification–mediated PCR (LAM-PCR) [17] on DNA cleaved with three different restriction enzymes (*Tsp*509I, *Hpy*CH4IV, *Hin*P1I). LAM-PCR products were pyrosequenced on a 454-Roche sequencer, yielding a total of 62,181 and 33,911 raw sequence reads from pre- and post-infusion T cells, respectively (available at the GenBank Short Read Archive (SRA) under the accession number SRA026258). Reads containing a complete retroviral sequence up to the CA dinucleotide were processed through an automated bioinformatic pipeline that eliminated small and redundant sequences and mapped the valid ones on the UCSC hg18 release of the human genome, to obtain 8,277 and 997 unique insertion sites from pre- and post-infusion T cells, respectively (**Table 1**). As a control dataset, we used 40,000 random genomic sites generated *in silico* taking into account the biases introduced by the LAM-PCR and pyrosequencing techniques (see Methods).

**Table 1:** Distribution of MLV integration sites in human T cells.

Source	Integrations	Intergenic	TSS-proximal	Intragenic	Forward orientation*	Reverse orientation*	Integration clusters	Cluster target genes**
TK38 pre infusion	6,044	2,031 (33.6%)	1,926 (31.9%)	2,087 (34.5%)	1,628 (50.7%)	1,584 (49.3%)		2,095
TK47 pre infusion	2,233	783 (35.1%)	610 (27.3%)	840 (37.6%)	608 (52.3%)	554 (47.7%)		1,201
Total	8,277	2,814 (34.0%)	2,536 (30.6%)	2,927 (35.4%)	2,216 (50.9%)	2,138 (49.1%)	1,362	3,296
TK38 post infusion	276	96 (34.8%)	71 (25.7%)	109 (39.5%)	52 (35.4%)	95 (64.6%)		69
TK47 post infusion	721	271 (37.6%)	199 (27.6%)	251 (34.8%)	131 (37.9%)	215 (62.1%)		133
Total	997	367 (36.8%)	270 (27.1%)	360 (36.1%)	183 (37.1%)	310 (62.9%)	102	212

**Figure 1:**
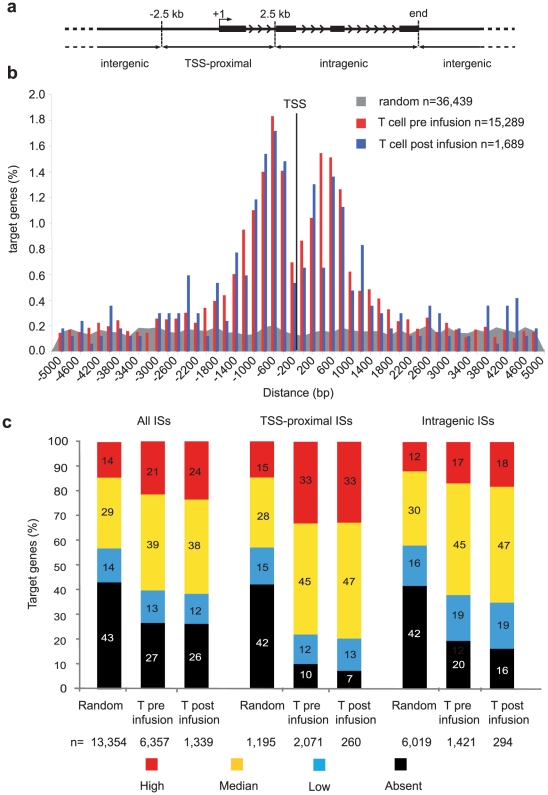
Genomic distribution and correlation with gene expression of MLV integration sites in human T cells. (**a**) MLV and random integration sites were annotated as TSS-proximal when located at ±2.5 kb from a transcription start site (TSS, +1) of a Known Gene (UCSC definition), intragenic when inside a gene at >2.5 kb from the TSS, and intergenic in any other case. Black bars represent exons of a schematic gene, arrowhead indicate the direction of transcription. (**b**) Distribution of the distance of MLV vector integrations from the transcription start site (TSS) of targeted genes in pre-infusion (red bars), and post-infusion (blue bars) T cells, at 200-bp resolution. The % of the total number of targeted genes (n) is plotted on the Y axis. The grey area indicates the distribution of control random sites. (**c**) Histogram distribution of expression values from an Affymetrix microarray (HG-U133 plus 2.0) analysis of RNA obtained from mock-transduced T lymphocytes. Affymetrix probe sets were re-annotated with custom CDF files [18] to obtain a single expression value for each gene. Expression levels were divided into four classes: absent (black portion of histogram bars), low (below the 25^th^ percentile of the normalized distribution, blue), intermediate (between the 25^th^ and the 75^th^ percentile, yellow) and high (above the 75^th^ percentile, red). The percentage distribution of the expression values of genes targeted by all integration/random sites (all ISs), TSS-proximal sites (TSS-proximal ISs) and intragenic sites (intragenic ISs) are shown by the left, middle or right group of bars, respectively. The number of genes (n) belonging to each category is indicated under the correspondent bar.

Distribution of MLV integration sites in the genome of pre- and post-infusion T cells from patients TK38 and TK47. Integrations were annotated for each patient as ‘TSS-proximal’ when occurring within a distance of ±2.5 kb from the TSS of at least one gene, as ‘intragenic’ when occurring into at least one gene at a distance of 2.5 kb from the TSS, and as ‘intergenic’ in all other cases (see Figure 1).

*Number of proviruses within introns or exons in forward or reverse orientation with respect to the direction of transcription.

**Number of genes having the TSS within ±50 kb from an integration cluster.

For all our analyses, we arbitrarily defined “target” genes all Known Genes having their transcription start site (TSS) within 50 kb from each integration/random site in either direction. In pre-infusion T cells, the SFCMM-3 vector integrated with a similar frequency inside and outside genes (48 *vs*. 52%, respectively, UCSC Known Genes track), with a significant over-representation of intragenic events when compared to the random control sites (48 *vs*. 38.5% p<10^−15^, two-sample test for equality of proportions with continuity correction). Integration sites were annotated as TSS-proximal when mapping in the ±2.5-kb window around a TSS, intragenic when mapping within a transcription unit >2.5 kb downstream a TSS, and intergenic in all other cases (**Figure 1A**). More than 30% of the integrations were TSS-proximal by this definition, with no significant difference between the two patiens (**Table 1**). We then plotted the distance between integration or random sites and the TSS of their target genes, at 200 bp resolution: 16.3% of the 15,289 target genes had an MLV insertion in the ±1,700 bp interval around the TSS, compared to a flat distribution observed for the random control sites (**Figure 1B**). The distribution showed a bimodal peak, with a drop in frequency at ±200 bp from the TSS. No significant difference was observed between pre-infusion and post-infusion T-cells both in the frequency of integrations within genes (48% *vs.* 49%) and in the proportion of genes harboring integrations around the TSS (16.3% *vs.* 14.5%, p>0.1 in all comparisons) (**Figure 1B**). A significant difference was instead observed in the orientation of the intragenic integrations between the two datasets. In pre-infusion T cells, 51.1% of the proviruses integrated within 4,374 genes were in forward transcriptional orientation, a virtually random distribution (p = 0.03). In post-infusion T cells, on the contrary, the majority (62.8%) of the proviruses integrated in 493 genes were in reverse orientation, a significantly biased distribution (p = 1.01×10^−15^) (**Table 1**), indicating a negative selection *in vivo* of cells carrying an intragenic, forward-oriented MLV provirus. The bias was even more pronounced for integrations occurring on the X chromosome, although their number was too small to allow a statistically significant comparison: 7 out of 8 (87.5%) X chromosome-specific, intragenic integrations were in reverse orientation in the post-infusion dataset *vs*. 28 out of 64 (44%) observed in pre-infusion cells.

To correlate MLV integration with the expression level of the target genes, we determined the expression profile of T cells activated with the same procedure used for transduction (mock-transduced) on Affymetrix HG-U133 plus 2.0 microarrays, run in triplicate. To ensure unequivocal probe-to-gene assignment, we re-annotated the HG-U133 plus 2.0 probe sets with custom chip definition files (CDF) [18], [19], where each of the 18,900 genes in the microarray is associated with a single, custom probeset that includes only probes unequivocally matching a transcript. More than 70% of the 6,357 analyzable genes targeted by MLV integration in pre-infusion T cells were scored as active at the time of transduction by Affymetrix analysis, compared to 57% of the genes hit by the random sites (p<10^−15^) (**Figure 1C**). Interestingly, >90% of the 2,071 genes harboring a TSS-proximal integration were scored as active, compared to 58% of the control dataset, with a significant over-representation (33%) of genes in the highest expression category (red in **Figure 1C**). These results confirm the general preference of MLV for active genes, and indicate that most of the targeted promoters are transcriptionally active at the time of transduction. The same correlations were observed in post-infusion T cells, with no significant overall or patient-specific differences compared to pre-infusion T-cells (**Figure 1C**).

### MLV integrates in transcriptionally active chromatin regions

**Figure 2:**
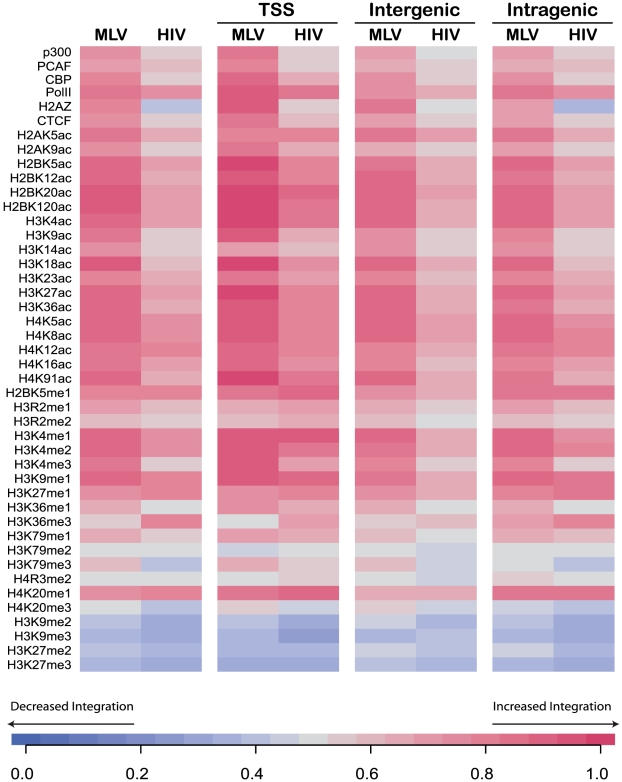
Association between histone modifications and retroviral integrations in T cells. Each row in the heat map corresponds to a different DNA-bound protein (p300, PCAF, CBP, Pol II, H2A.Z, CTCF) or histone post-translational modification (acetylation, ac, and methylation, me), according to the ChIP-seq databases from Barski *et al*. [20] and Wang *et al*. [21]. Chromatin features were annotated in an interval of ±500 bp around MLV and HIV vector integrations, as whole datasets or split into the three classes reported in Figure 1a. Color shades from blue to red are indicative of the direction and the strength of the association between epigenetic features and integrations, as calculated by statistical comparison against matched random controls using the ROC area method [23]. ROC values between 0 and 0.5 (blue shades) reflect a negative correlation compared to random, 0.5 (grey) means no correlation, values above 0.5 (red shades) indicate a positive correlation. Results of statistical tests comparing data sets to each other and to random can be found in the Supplementary statistical analysis S1.

To gain insight on the chromatin conformation of the genomic regions targeted by MLV in T cells, we annotated the 8,277 integration sites in pre-infusion T cells with 41 types of histone modifications (methylations and acetylations) or chromatin-bound proteins mapped genome-wide in human T cells [20], [21]. As a comparison, we re-analyzed 7,782 insertions of an HIV-derived lentiviral vector in pre-infusion T-cells [22]. The analysis was carried out in a window of 1 kb (±500 bp) around each insertion site, using matched random controls (see Methods for definition) as background. The direction and strength of the correlations between epigenetic features and retroviral integrations were quantified using the Receiver Operation Characteristic (ROC) method as previously described [23], allowing the associations to be graphically expressed as heat maps. Integrations were analyzed as a whole or broken down into TSS-proximal, intragenic and intergenic groups. As shown in **Figure 2**, histone modifications marking active transcription units and/or enhancers (all acetylations and H3K4 methylations) showed the strongest association (red shades in the heat map) with MLV integration sites. The association was particularly evident for, but not limited to, TSS-proximal insertions. A similar scenario was found for the binding of the p300 and CBP histone acetyl transferases, Pol II, the insulator-binding protein CTCF and the H2A.Z histone variant. A weaker enrichment of transcription-associated histone modifications was generally observed around HIV insertion sites, with the exception of those extending throughout the body of active genes (H4K12ac, H2BK5me1, H3K27me1, H3K36me3, and H4K20me1). H2A.Z was under-represented around HIV insertion sites compared to matched random controls. Both MLV and HIV integrations were negatively associated (blue shades in the heat map) with marks linked to transcriptional repression and/or heterochromatin (H3K27 and H3K9 mono- and di-methylations). Finally, little or no correlation with retroviral integration was scored for histone modifications that have no evident bias towards active or inactive genes (H3K79 methylations, H3R/H4R methylations and H4K20me1) (**Figure 2** and **Supplementary Statistical Analysis S1**).

### MLV integrations are clustered and target genes involved in T-cell functions

A plot of the distance between consecutive integrations in the T-cell genome showed that MLV integration sites were highly clustered compared to the random integration data set, both in pre- and post-infusion cells (**Figure S1**). A statistical comparison of the distribution of MLV and random integrations indicated a threshold of significance (p<0.01) for cluster definition of 2 integrations within 3,308 bp for pre-infusion and 27,230 bp for post-infusion T cells, respectively (see legend of **Figure S1**). By applying these thresholds, we identified 1,362 clusters containing 2 to 11 integrations in pre-infusion T cells and 102 clusters containing 2 to 6 integrations in post-infusion T cells, targeting 2,157 and 160 genes respectively. There was no significant difference in the relative frequency of clusters between pre- and post-infusion T cells, as determined by sampling ten times the pre-infusion dataset in order to compare distributions with the same numerical complexity (two-sample Kolmogorov-Smirnov test, p = 0.66 not shown).

**Figure 3:**
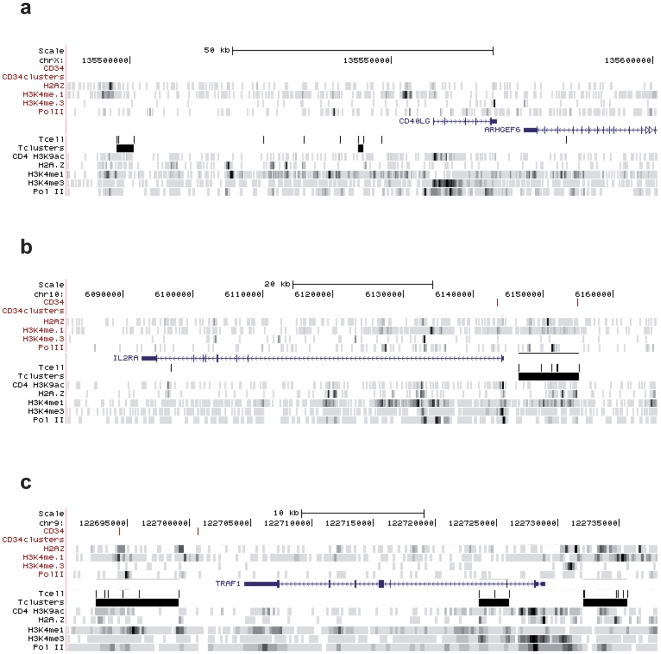
MLV integration clusters in T cell-specific loci. Distribution of MLV integrations (black bars for T cells and red bars for CD34^+^ hematopoietic progenitor cells), and clusters (black boxes for T cell and red boxes for CD34^+^ cells), within three RefSeq gene loci (CD40LG, IL2RA, TRAF1) specifically expressed in T cells, as displayed by the UCSC genome browser. H2A.Z, H3K4me1, H3K4me3, H3K9ac and Pol II tracks are those determined by ChIP-seq in the genome of human CD34^+^/CD133^+^ HPCs [36] (in red, above the gene) and in human primary T cells [20] (in black, below the gene). Blue boxes represent exons, arrowheads in introns indicate the direction of transcription.

**Figure 4:**
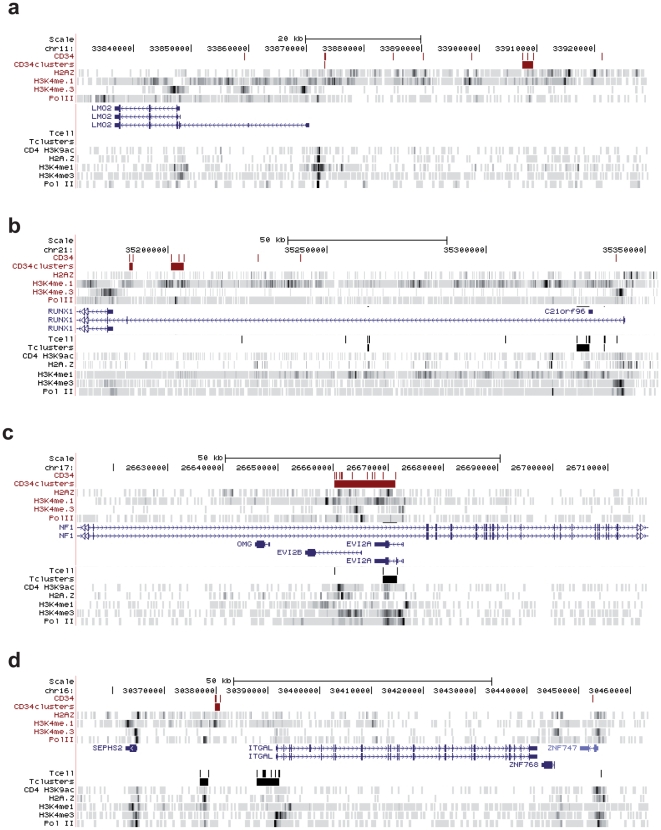
MLV integration clusters in CD34^+^ HPC-specific loci. Distribution of MLV integrations and clusters within three RefSeq gene loci expressed in both T cells and CD34^+^ cells (RUNX1, EVI2A/B, ITGAL) or only in CD34^+^ HPCs (LMO2), as displayed by the UCSC genome browser. See legend of Figure 3 for explanation of symbols.

When analyzed at single-locus resolution, MLV integration clusters in pre-infusion T-cells appear to co-map with promoters and putative regulatory regions of expressed genes. **Figure 3** shows three examples of such associations. A ∼50-kb region upstream of the TSS of the CD40LG gene contained 2 clusters (black bars in **Figure 3A**) and a total of 9 integrations flanking peaks of histone modifications associated with enhancer function (H3K4me1) and upstream of the CD40LG promoter, identified by peaks of H3K4me3, H3K9ac and Pol II. Similarly, the promoter of the T cell-specific IL2RA gene, marked by H3K4me3 H3K9ac and Pol II, is flanked by a cluster of 6 MLV integrations, marked by peaks of H3K4me1 and the H2A.Z variant (**Figure 3B**). Three clusters containing a total of 15 integrations were identified in less than 40 kb in the TRAF1 locus, upstream, within and downstream of the transcription unit (**Figure 3C**). Again, the clusters were associated with peaks of H3K4me1, H3K9ac and H2A.Z, identifying putative regulatory regions in the locus. The MLV integration peaks appeared to be cell-specific, since no clusters and very few, sporadic integrations were identified in the same regions in a collection of 8,000 integrations mapped in the genome of human cord blood CD34^+^ HPCs (Cattoglio *et al*., submitted), characterized by a different pattern of histone modifications (**Figure 3**). Conversely, regions heavily targeted by integration clusters in the genome of CD34^+^ HPCs, such as the LMO2 locus, contained no integration in the T-cell genome (**Figure 4A**). In loci expressed in both cell types, such as RUNX1, EVI2A/B, and ITGAL, integration clusters were localized in different regions in HPCs (red bars) and T cells (black bars), co-mapping with cell-specific histone modifications associated to promoters and regulatory regions (**Figure 4B–D**).

**Figure 5:**
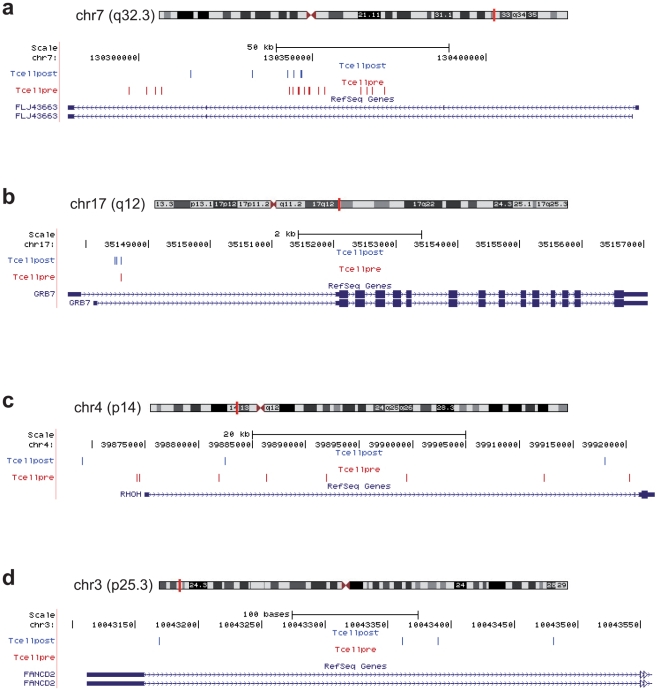
Retroviral integration clusters in pre- and post-transplantation T cells. Representation of four clusters of MLV integrations in pre-infusion (red bars) and post-infusion (blue bars) T cells, mapped by the UCSC genome browser. Location of the clusters is indicated by a vertical red bar on the corresponding chromosome (top). Zooming-in the UCSC window, the base position feature (scale bar and nucleotide number) identifies the genomic coordinates of the displayed region. The RefSeq genes track shows known human protein-coding transcripts taken from the NCBI RNA reference sequences collection. Blue boxes represent exons, arrowheads indicate the direction of transcription.

Interestingly, 58% of the 102 clusters identified in post-infusion T cells targeted genes that were targeted also in the pre-infusion dataset (**Table S1**), indicating that the clustered distribution is mostly determined by MLV integration preferences and not by selection *in vivo*. **Figure 5A–C** shows three examples of overlapping integration clusters in pre-infusion (red) and post-infusion (blue) T cells in the FLJ43663, GRB7, and RHOH loci. The only exception was the FANCD2 locus with a cluster of 4 integrations in 312 bp present only in post-infusion T cells from patient TK38 (**Figure 5D**). Ten integrations were mapped at exactly the same nucleotide in pre- and post-infusion T cells from patient TK47 (**Table S2**), most likely indicating the presence of over-represented cell clones in both samples. No such event was observed for patient TK38, for which more integrations were retrieved in the pre-infusion sample (6,044 *vs*. 2,233 in TK47) and less in post-infusion cells (276 *vs*. 721). This difference may reflect the different numbers of TK^+^ cells circulating in the two patients at the time of harvesting.

**Figure 6:**
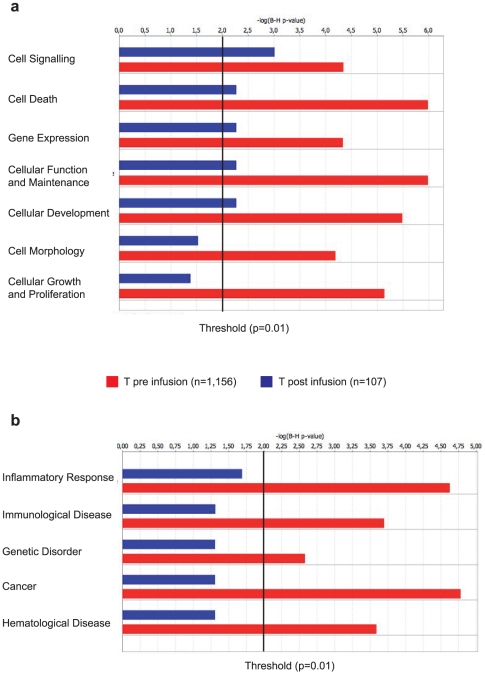
Functional classification of genes targeted by integration clusters. The figure shows those function (**a**) and disease (**b**) categories significantly over-represented among the target genes of MLV integration clusters in pre-infusion (red bars) or post-infusion (blue bars) T cells, as analyzed by the Ingenuity® software, using the Ingenuity Pathways Knowledge Base gene population as background. P values were corrected for multiple testing by the Bonferroni method. Statistical significance was set at a p-value of 0.01 (2 in log scale, vertical line). (n) represents the total number of genes eligible for the analysis.

A functional classification of the genes annotated within the integration clusters was carried out by the Ingenuity® software. Functional categories significantly over-represented in the pre-infusion dataset included cell signaling, growth and proliferation, development and death (red bars in **Figure 6A**), all above the threshold of statistical significance (black vertical line in **Figure 6A**) compared to the Ingenuity Knowledge Base background. Over-represented disease-associated categories included inflammatory response, immunological, hematological and genetic disorders (**Figure 6B**), well related to T cell-specific functions. All these categories were barely or not at all over-represented in the post-infusion dataset, indicating no preferential survival *in vivo* of cells harboring integrations in specific gene categories, particularly cell growth and proliferation and cancer (blue bars in **Figure 6**). To investigate the association with oncogenic transformation more directly, we annotated all cancer-related genes targeted by MLV integration clusters, using a comprehensive compilation that includes proto-oncogenes and genes associated with common insertion sites (CIS) in murine tumors. Genes cumulatively belonging to both categories accounted for 11.7 and 16.0% of the genes targeted by the clusters in pre- and post-infusion T cells respectively, a significant over-representation with respect to the frequency observed in the random integration dataset (7.8% of 15,200 genes, p<10^−4^). However, the difference between pre- and post-integration datasets was not significant (p = 0.11) and 71% of the cancer-associated genes targeted by post-infusion clusters (in bold in **Table S1**) were targeted also by pre-infusion ones, suggesting no selection *in vivo* for cells harboring integrations around such genes.

**Figure 7:**
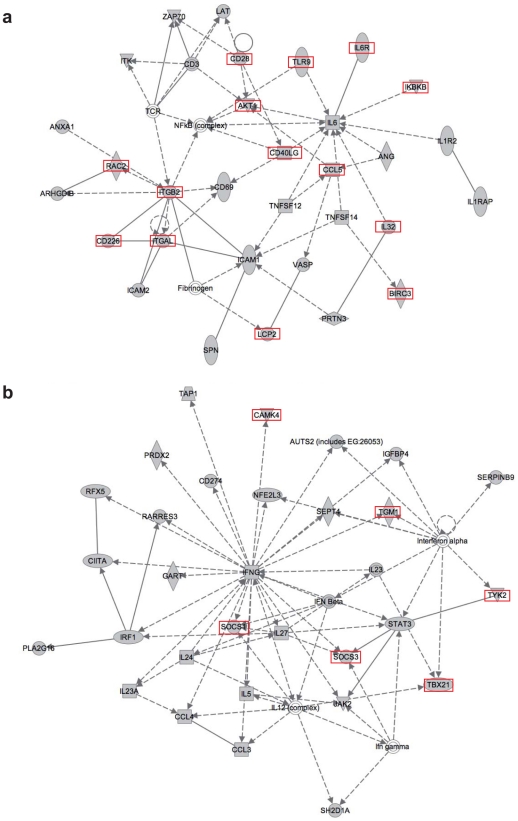
Genes targeted by MLV integration clusters are functionally linked in gene networks. Networks originated by Ingenuity® analysis of 31 (**a**) and 30 (**b**) eligible T cell pre-infusion cluster targets. The most relevant networks, having a score of 21 (p<10^−14^ for all top functions associated to the network **a**) and 19 (network **b** p<10^−8^) are indicated. Red boxes indicate the 14 (**a**) and 6 (**b**) Focus Genes in common with the post-infusion dataset.

An Ingenuity functional network analysis performed on the 2,157 genes targeted by pre-infusion T cells clusters displayed two main networks, containing 30 and 31 Focus Genes in the categories of inflammatory response (e.g., IL6, CD40LG, ITGAL) and immunological diseases (e.g., IFNγ, IFNβ, IL27) respectively (**Figure 7**). Only 14 and 6 of the Focus Genes in the two networks were targeted by MLV integration in post-infusion cells (boxed in red in **Figure 7**). They were not node genes of the network and were not targeted by post-infusion integration clusters, except for two T cell-specific genes: CD40LG, highly expressed in T cells and crucial for their crosstalk with dendritic and B cells, and ITGAL that plays a role in lymphocytes co-stimulatory signaling.

## Discussion

Integration of MLV-derived retroviral vectors may have significant consequences on gene expression and homeostasis of transduced and transplanted target cells, particularly in the hematopoietic system. The enhancer activity of the MLV LTRs may de-regulate proto-oncogenes, and cause pre-neoplastic clonal expansion [10], [11], leukemic transformation without clonal expansion [8], [9], [24], or no apparent adverse effect [13] depending on the disease context and a number of still ill-defined factors. Integration sites can be used as markers of clonality to study the clonal dynamics of transduced cells *in vivo*, and provide important clues to predict the potential genotoxicity of MLV integration in a specific cell or disease context [12], [22], [24], [25], [26].

We used LAM-PCR and pyrosequencing to derive a high-definition map of MLV integration sites in the genome of human T cells, before and after infusion in two patients treated with DLI after haploidentical HSC transplantation for high-risk leukemia. The analysis of >8,000 integrations in pre-infusion T cells showed a clustered distribution of integration sites around the TSS of active genes and, in general, around genomic regions characterized by epigenetic signatures associated to active promoters and enhancers. These included acetylations of H2A, H2B, H3 and H4, methylations of H3 (H3K4me1, H3K4me2, H3K4me3), and binding of the p300 and CBP histone acetil transferases, normally associated to enhancers and promoters, Pol II, CTCF and H2AZ, a histone variant enriched at targets of the Polycomb complex that establishes specialized chromatin domains and plays a crucial role in the regulation of cell commitment and differentiation [27]. Interestingly, most of these associations are statistically significant for all integrations, whether they are TSS-proximal, intragenic or intergenic ones, indicating that MLV PICs are attracted to regions marked by specific epigenetic modifications independently from their location around TSSs. Many of these modifications are indeed specific for MLV integration, as they are either poorly enriched (e.g., H3K9ac, H3K14ac, H3K4me3, p300 and CBP) or under-represented (H2A.Z) around a comparable number of HIV-derived, lentiviral vector integrations in T cells. HIV proviruses are instead preferentially associated to histone modifications extending throughout the body of active genes (H4K12ac, H2BK5me1, H3K27me1, H3K36me3, and H4K20me1), consistent with the integration preferences of lentiviral vectors [22], [28].

The tendency of MLV to integrate near gene promoters has been reported previously [14]. However, fine mapping of MLV sites around the TSS of >2,400 genes in T cells showed a bimodal distribution, with fewer insertions in the −200 to +200 region, where the general transcription factors contact the core promoter and recruit RNA Pol II [29]. This suggests that most of the promoters targeted by MLV PICs are engaged by the basal transcriptional machinery, acting as a physical obstacle to integration. Consistently, an analysis of the transcription profile of mock-transduced T cells showed that 90% of the genes targeted by MLV in the ±2.5-kb region around the TSS are active at the time of transduction.

Analysis of the distribution of insertion sites shows that MLV integration is highly clustered in the T cell genome, with peaks of integrations within apparently highly preferred regions. The statistical definition of an integration cluster was a critical aspect of this study. We abandoned the classical definition of “common integration site” originally developed to define integration-associated oncogenes [30], and adopted a new definition statistically modeled on the size of the dataset. To define the minimal distance between two or more integrations in a cluster, we used a procedure that entails a variable threshold, proportional to the total number of integration sites, allowing direct comparison between datasets of different size. Applied to >8,000 integration sites in pre-infusion T cells, the procedure identified >1,350 clusters, containing 2 to 11 sites and targeting 3,296 genes. Functional clustering analysis carried out with the Ingenuity platform indicates that genes targeted by MLV clusters are regulated during T cell development and differentiation, and are linked in functional networks that play essential roles in T cell function. These include genes associated to cancer by most classifications, suggesting that they are targeted because of their biological rather than oncogenic function. Similar correlations were already reported for genes targeted by MLV in CD34^+^ HPCs [15], indicating that MLV integration is associated to gene expression programs in a cell-specific fashion. Analysis of single loci showed that genes are targeted by clusters only in the cells in which they are expressed (e.g., IL2RA in T cells and LMO2 in CD34^+^ HPCs), where they co-map with promoters and known or putative enhancers identified by peaks of H3K4me3, H3K4me1, H2A.Z and Pol II. Interestingly, genes expressed in both cell types are either targeted at the same sites (e.g., EVI2A/B) or at different, cell-specific ones, co-localizing with cell-specific peaks of histone modifications and Pol II binding (e.g., RUNX1). This suggests that MLV PICs may be targeted to differentially used promoters and enhancers, engaged by the Pol II transcriptional machinery in a cell-specific fashion.

An important aspect of this study was the use of integration sites as lineage tracers to analyze the clonal dynamics of infused T cells *in vivo*. The majority of the T cells that reconstitute the immune system in TK^+^ DLI-treated patients are TK^−^ cells, while TK^+^ cells are rapidly diluted in the periphery unless they expand for a specific reason (e.g., GvHD) [3], [4]. We could therefore analyze clonal dynamics in only a minority of patients, who retained enough TK^+^ cells (>5%) in peripheral blood two months after infusion to allow their sorting and extraction of a sufficient amount of genomic DNA. The comparative analysis of integration sites in pre-infusion and post-infusion T-cells showed very similar patterns in both populations in terms of proportion of intragenic *vs*. intergenic and TSS-proximal sites, distribution around the TSSs, correlation with gene expression levels and relative abundance of clusters. In fact, most of the clusters identified in post-infusion T cells (102, targeting 160 genes) were observed also in pre-infusion cells, indicating that they were generated by the MLV integration preferences for certain genomic regions rather than by *in vivo* selection. We did not observe significant patient-specific differences in any of the parameters utilized to analyze pre- vs. post-infusion cells. Analysis of the biological functions associated to the genes targeted by the clusters actually showed little or no over-representations of proto-oncogenes or genes associated with signal transduction and cell proliferation in post-infusion T cells, and no integrations in the genes most crucial for the functional networks that were over-represented in pre-infusion cells. These data provide little evidence for clonal expansion or selection of cells harboring integrations in growth-controlling or otherwise “dangerous” genes. On the contrary, they suggest selective loss of cells harboring integrations that may affect general or T cell-specific functions.

The selective post-infusion loss of cells harboring proviruses in direct transcriptional orientation within introns or exons is particularly striking. Forward-oriented proviruses insert the MLV splicing and polyadenylation signal in the primary transcripts, and are likely to cause gene inactivation by aberrant splicing and/or premature termination in a number of cases, with consequent loss of gene function. These events are clearly counterselected *in vivo*, indicating that clonal loss, rather than dominance, is the most frequent genotoxic consequence of MLV transduction for T cells. This hypothesis is supported by the even stronger counterselection observed for forward-oriented proviruses in genes active on only one allele, as those located on the X chromosome. Orientation biases were previously observed for endogenous retroviruses [31], [32], indicating that gene-inactivating integration events are counterselected during evolution of the host genomes. The loss of T-cell clones observed in this study is expected to be random and quantitatively modest, and appears to have no consequences on the size and functionality of the donor T cell immune repertoire, and most importantly, on the efficacy of DLI after allogeneic HSC transplantation [3], [4]. This is further supported by the spectratype of TCR Vβ families observed on TK^+^ cells sorted from the two patients. The analysis, revealed a wide TCR repertoire, with 23 out of 24 TCR-Vβ families represented, each showing a wide, oligoclonal profile (not shown).

High-definition maps of retroviral integration sites are a powerful tool to analyze the fate of genetically modified cells upon administration to patients. Pre-infusion maps provide expected targeting frequencies for gene loci and for specific elements within each locus. Comparing these frequencies with those observed *ex vivo* in follow-up studies allows to monitor any clonal imbalance and possibly predict adverse events caused by pre-malignant expansion of cells carrying gene deregulating insertions. Overall, our study indicates the absence of substantial genotoxic risks in the use of TK-modified T cells, confirming the clinical evidence of absence of treatment-related adverse events in over 50 patients treated over more than ten years [3], [4], [5], [6], and the experimental evidence of the relative resistance of T cells to oncogene transformation compared to hematopoietic stem and progenitor cells [33].

## Materials and Methods

### Patients and cells

The use of the TK DLI in the context of allo-HSC transplantation was approved by the Italian Ministry of Health. Orphan drug status has been granted by the European Medicine Agency (EMA). The study, sponsored by Molmed S.p.A. (www.molmed.com), was approved by the Institutional Review Board of the San Raffaele Scientific Institute and informed written consent was obtained from donors and recipients. Patients TK38 and TK47 were treated with HLA-haploidentical HSC transplantation for high-risk acute myeloid leukemia, in remission at time of transplantation [4]. They received one infusion of 10^7^/kg of T cells transduced with the SFCMM-3 retroviral vector, encoding HSV-TK and ΔLNGFR [1], [34], as previously described [3], [4]. TK-positive lymphocytes were selected after transduction to >90% purity by immunomagnetic sorting for ΔLNGFR expression, and stored frozen up to the day of infusion. PBMCs were obtained 65 (TK38) and 62 (TK47) days after infusion by Ficoll-Hypaque gradient separation, sorted for ΔLNGFR expression, and expanded in culture with OKT3, irradiated feeders and IL2. At time of T cell harvesting, we detected 262 and 654 LNGFR^+^ cells/mcl in TK38 and TK47, representing respectively 11.4% and 30.2% of circulating CD3+ lymphocytes. Both patients experienced moderate-grade GvHD, which was controlled by administration of Ganciclovir. At the time of sampling, both patients were in complete remission, free from GvHD. T cells were harvested before ganciclovir treatment.

### Sequencing, mapping and annotation of retroviral integration sites

Genomic DNA was extracted and digested with three “four cutter” enzymes: *Tsp*509I, *Hpy*ChIV4, *Hin*P1. 5′-LTR vector-genome junctions were amplified by LAM-PCR as previously described [17]. A further PCR step was performed to include sample specific barcodes for parallel sequencing using the 454/Roche pyrosequencing platform [35]. Sequence reads were processed through an automated bioinformatic pipeline that eliminated small and redundant sequences and mapped the valid ones on the UCSC hg18 release of the human genome. Valid reads, contained the LTR sequence up to the CA integration dinucleotide and 20 bp or more of human genomic sequence were used to generate a non-redundant dataset using the nrdb tool (available at http://www.advbiocomp.com/blast.html in the AB-BLAST software package). Non-perfectly redundant reads were than mapped onto the human genome, requiring the alignment to start within the first three nucleotides and to possess a minimum of 90% identity. Sequences were discarded as mapping to multiple sites when they had more than one match on the human genome differing in identity less than 2%. Identity for Blat was calculated as follows: [matching nt - (mismatching nt + query gap + tile gap)]/query size. All UCSC Known Genes having their transcription start site (TSS) at ±50 kb from an integration or random site were annotated as targets. In case of multiple transcript variants, we arbitrarily chose the isoform with the nearest TSS to an integration or random site. A matched control set of 40,000 random sites was generated *in silico* on the human genome by discarding sites with the nearest *Tsp*509I, *Hpy*ChIV4, or *Hin*P1 recognition site at <20 bp (the minimum requirement for a blast search) or >500 bp (the maximum estimated length for efficient 454 bead loading). Integration clusters were statistically defined as described in the legend of **Figure S1**.

### Gene expression profiling

The expression profile of T lymphocytes was determined by microarray analysis of cells isolated from healthy donor PBMCs, and activated for 72 hrs under the same condition used for transduction (mock transduced). RNA was extracted from 1–2×10^6^ cells, transcribed into biotinylated cRNA, hybridized to Affymetrix HG-U133A plus 2.0 Gene Chip arrays and analyzed as previously described [6], [15]. The arrays were re-annotated using a set of previously described custom CDFs and the corresponding Bioconductor libraries [18], [19] based on the GeneAnnot database that compares all Affymetrix probes with transcript sequences from publicly available cDNAs, GenBank, RefSeq and Ensembl repositories. To correlate retroviral integration and gene activity, average expression values from the three microarrays were divided into four classes, *i.e.*, absent, low (below the 25^th^ percentile in a normalized distribution), intermediate (between the 25^th^ and the 75^th^ percentile) and high (above the 75^th^ percentile).

### Functional annotation and clustering analysis

Functional annotation of genes targeted by integration clusters were performed by the Ingenuity Pathways Analysis (IPA) tool (Ingenuity Systems, www.ingenuity.com). Genes from the dataset associated with biological functions and/or diseases in the Ingenuity Pathways Knowledge Base (IPKB) were considered for the analysis. Fischer's exact test with Bonferroni correction for multiple testing was used to calculate a p-value determining the probability that each biological function and/or disease assigned to that data set is due to chance alone. A data set containing gene identifiers was uploaded into in the IPA application. Each gene identifier was mapped to its corresponding gene object in the IPKB. These genes (Focus Genes) were overlaid onto a global molecular network developed from information contained in the IPKB. Networks were algorithmically generated based on the direct or indirect interaction between the sole Focus Genes. The Functional Analysis of each network identified the biological functions and/or diseases that were most significant to the genes in the network (Fischer's exact test with Bonferroni correction for multiple testing). Cancer-associated genes were annotated using a compiled library of proto-oncogenes and genes associated with common insertion sites in murine tumors (http://microb230.med.upenn.edu/protocols/cancergenes.html).

### Bioinformatic analysis of associations with epigenetic modifications

To correlate histone modifications and retroviral integration frequency, a window of ±500 bp around each insertion site was annotated with a number of histone methylation/acetylation sites or bound chromatin proteins obtained from publicly available ChIP-seq data in human primary T cells [20], [21]. The distance was calculated by subtracting the position of the integration from the midpoint of the epigenetic feature. For comparison in the statistical analysis, we also generated sets of matched random controls. For this, a large library of random sites was generated, and then the distances to restriction enzyme recognition sites scored. Each experimental site was matched with 3 control sites that were positioned the same number of bases from a restriction site as for the experimental site. Statistical comparisons were calculated using the R function “ROC.strata” programmed by Chuck Berry [23]. The program takes epigenetic feature counts for each insertion site and the respective matched random controls to obtain a value between 0 and 1, representing a positive or negative correlation between each integration and that particular feature. These values are then averaged over the entire dataset to obtain a ROC value for a given epigenetic feature versus an integration site dataset (see Supplementary statistical analysis for complete lists of ROC and P values).

## Supporting Information

Statistical Analysis S1 Figure 2 presents a heat map summarizing the relationship of density of sites of epigenetic modification to sites of integration. The color code shows the frequency of each type of modification compared to chance (i. e. random control sites). This rar file contains an interactive version of the heat map shown in Figure 2 that allows visualization of the results of statistical tests. After unzipping the file, open the file named main.svg in Safari. Clicking on the heading for each row calls up statistical tests for comparisons to other rows. Clicking on each column calls up tests for each column. Clicking on “compare to area = 0.05” calls up tests for comparison to random integration. * indicates 0.05>P>0.01; ** indicates 0.01>P>0.001; *** indicates P<0.001. A summary of the methods used for statistical testing can be found in Berry C, et al., PLoS Comput Biol 2: e157.
(RAR)

Figure S1 **Statistical definition of retroviral integration clusters.** To establish a statistically rigorous definition of integration cluster, we plotted the percentage distribution of the distances (log scale on the *x* axis) between any MLV insertion site (S1) and the first consecutive site (S2) in pre-infusion (red line, left panels) and post-infusion (red line, right panels) T cells, together with the same number of control random sites (black lines in both panels), re-sampled 1,000 times from a collection of 40,000 random sites. Dashed vertical lines identify the threshold in the control site distribution containing 1% or 5% of the sites, representing the false discovery rate (FDR) for cluster definition. With a FDR of 1%, the threshold for defining a cluster was two integration sites in 3,308 bp for the pre-infusion and 27,230 bp for the post-infusion dataset.
(EPS)

Table S1 **Integration clusters in post-infusion T cells.** The Table show 102 integration clusters identified in post infusion T cell. For each cluster, the table reports the number of hits determining the cluster (cluster dimension), the source (patient), the genomic position (chromosome, position start and end), the target genes (gene symbol and entrez gene) and the genes in common with clusters identified in pre-infusion T cells. Cancer-associated genes (defined in http://microb230.med.upenn.edu/protocols/cancergenes.html) are indicated in bold.
(DOC)

Table S2 **Identical integration in pre- and post-infusion T cells from patient TK47.** The Table shows ten integrations mapping exactly at the same nucleotide in pre- and post-infusion T cells from patient TK47. For each integration, the table reports the genomic position, the annotation (TSS-proximal, intragenic or intergenic, see legend of Figure 1) and the target gene (gene symbol and entrez gene).
(DOC)
