# Supplementary material for: High-Definition Mapping of Retroviral Integration Sites Defines the Fate of Allogeneic T Cells After Donor Lymphocyte Infusion
Source: PLoS One. 2010 Dec 22;5(12):e15688. doi: 10.1371/journal.pone.0015688 (PMC3008730; doi:10.1371/journal.pone.0015688)
Supplement: Table S1 — Integration clusters in post-infusion T cells. The Table show 102 integration clusters identified in post infusion T cell. For each cluster, the table reports the number of hits determining the cluster (cluster dimension), the source (patient), the genomic position (chromosome, position start and end), the target genes (gene symbol and entrez gene) and the genes in common with clusters identified in pre-infusion T cells. Cancer-associated genes (defined in http://microb230.med.upenn.edu/protocols/cancergenes.html) are indicated in bold. (DOC) [file pone.0015688.s003.doc]

**Table S2. Identical integrations in pre- and post- infusion T cells from patient TK47**

| ID | CHROMOSOME | POSITION | ENTREZ GENE | ANNOTATION | GENE SYMBOL |
| --- | --- | --- | --- | --- | --- |
| 1 | 11 | 6367901 | 6609 | TSS-proximal | SMPD1 |
| 2 | 7 | 17145709 |  | intergenic |  |
| 3 | 20 | 23328385 | 63908 | intragenic | NAPB |
| 4 | 6 | 26325863 | 3013 | TSS-proximal | HIST1H2AD |
|  |  |  | 8339 | TSS-proximal | HIST1H2BG |
|  |  |  | 8345 | TSS-proximal | HIST1H2BH |
|  |  |  | 8351 | TSS-proximal | HIST1H3D |
|  |  |  | 8353 | TSS-proximal | HIST1H3E |
|  |  |  | 8360 | TSS-proximal | HIST1H4D |
|  |  |  | 8361 | TSS-proximal | HIST1H4F |
| 5 | 17 | 35148562 | 2886 | TSS-proximal | GRB7 |
| 6 | 15 | 36739911 |  | intergenic |  |
| 7 | 13 | 59870565 | 81550 | TSS-proximal | TDRD3 |
| 8 | 5 | 61635462 | 3796 | TSS-proximal | KIF2A |
| 9 | 5 | 96237709 | 64167 | TSS-proximal | ERAP2 |
| 10 | 14 | 104354210 |  | intergenic |  |

The Table show ten integrations mapping exactly at the same nucleotide in pre- and post-infusion T cells from patient TK47. For each integration, the table reports the genomic position, the annotation (TSS-proximal, intragenic or intergenic, see legend of Figure 1) and the target gene (gene symbol and entrez gene).
