# Supplementary material for: High-Definition Mapping of Retroviral Integration Sites Defines the Fate of Allogeneic T Cells After Donor Lymphocyte Infusion
Source: PLoS One. 2010 Dec 22;5(12):e15688. doi: 10.1371/journal.pone.0015688 (PMC3008730; doi:10.1371/journal.pone.0015688)
Supplement: Table S2 — Identical integration in pre- and post-infusion T cells from patient TK47. The Table shows ten integrations mapping exactly at the same nucleotide in pre- and post-infusion T cells from patient TK47. For each integration, the table reports the genomic position, the annotation (TSS-proximal, intragenic or intergenic, see legend of Figure 1) and the target gene (gene symbol and entrez gene). (DOC) [file pone.0015688.s004.doc]

**Table S1. Integration clusters in post-infusion T cells**

| CLUSTER ID | CLUSTER DIMENSION | PATIENT | CHROMOSOME | POSITION START | POSITION END | GENE SYMBOL | ENTREZ GENE | GENES IN COMMON IN PRE-INFUSION  T CELLS |
| --- | --- | --- | --- | --- | --- | --- | --- | --- |
| 1 | 2 | TK 47 | 1 | 16155317 | 16155318 | ZBTB17 | 7709 | TK 38 |
|  |  | TK 47 |  |  |  | **C1orf64** | 149563 |  |
| 2 | 2 | TK 47 | 1 | 59546288 | 59552337 | FGGY | 55277 |  |
|  |  | TK 47 |  |  |  |  |  |  |
| 3 | 2 | TK 47 | 1 | 92813508 | 92816268 | EVI5 | 7813 | TK 38, TK 47 |
|  |  | TK 47 |  |  |  |  |  |  |
| 4 | 2 | TK 47 | 1 | 148116488 | 148126630 | HIST2H2AC | 8338 |  |
|  |  | TK 47 |  |  |  | HIST2H2BE | 8349 |  |
|  |  |  |  |  |  | SV2A | 9900 |  |
|  |  |  |  |  |  | SF3B4 | 10262 |  |
|  |  |  |  |  |  | BOLA1 | 51027 |  |
|  |  |  |  |  |  | HIST2H2AB | 317772 |  |
|  |  |  |  |  |  | HIST2H3C | 333932 |  |
|  |  |  |  |  |  | HIST2H4A | 554313 |  |
|  |  |  |  |  |  | HIST2H2AA3 | 723790 |  |
|  |  |  |  |  |  | MTMR11 | 10903 |  |
| 5 | 2 | TK 47 | 1 | 154985350 | 154987536 | CRABP2 | 1382 | TK 38 |
|  |  | TK 47 |  |  |  | HDGF | 3068 | TK 38 |
|  |  |  |  |  |  | **PRCC** | 5546 | TK 38 |
|  |  |  |  |  |  | C1orf66 | 51093 | TK 38 |
|  |  |  |  |  |  | MRPL24 | 79590 | TK 38 |
|  |  |  |  |  |  | ISG20L2 | 81875 | TK 38 |
| 6 | 3 | TK 47 | 1 | 196847114 | 196873477 | PTPRC | 5788 | TK 38 , TK 47 |
|  |  | TK 47 |  |  |  |  |  |  |
|  |  | TK 47 |  |  |  |  |  |  |
| 7 | 3 | TK 47 | 1 | 211290874 | 211291121 | RPS6KC1 | 26750 |  |
|  |  | TK 38 |  |  |  | ANGEL2 | 90806 |  |
|  |  | TK 47 |  |  |  |  |  |  |
| 8 | 2 | TK 47 | 1 | 233182511 | 233189047 | NA |  |  |
|  |  | TK 47 |  |  |  |  |  |  |
| 9 | 2 | TK 47 | 1 | 242550263 | 242552777 | C1orf100 | 200159 |  |
|  |  | TK 47 |  |  |  |  |  |  |
| 10 | 3 | TK 38 | 2 | 25898566 | 25907364 | ASXL2 | 55252 |  |
|  |  | TK 38 |  |  |  |  |  |  |
|  |  | TK 47 |  |  |  |  |  |  |
| 11 | 2 | TK 38 | 2 | 48396182 | 48398153 | FOXN2 | 3344 |  |
|  |  | TK 47 |  |  |  |  |  |  |
| 12 | 2 | TK 47 | 2 | 162515926 | 162531461 | SLC4A10 | 57282 | TK 47 |
|  |  | TK 47 |  |  |  |  |  |  |
| 13 | 2 | TK 38 | 2 | 196750707 | 196757665 | STK17B | 9262 | TK 38 , TK 47 |
|  |  | TK 38 |  |  |  |  |  |  |
| 14 | 2 | TK 38 | 2 | 222089922 | 222089928 | EPHA4 | 2043 |  |
|  |  | TK 38 |  |  |  |  |  |  |
| 15 | 2 | TK 38 | 2 | 231230355 | 231241108 | CAB39 | 51719 | TK 38 , TK 47 |
|  |  | TK 47 |  |  |  |  |  |  |
| 16 | 4 | TK 38 | 3 | 10043169 | 10043481 | **FANCD2** | 2177 |  |
|  |  | TK 38 |  |  |  | TMEM111 | 55831 |  |
|  |  | TK 38 |  |  |  | LOC401052 | 401052 |  |
|  |  | TK 38 |  |  |  |  |  |  |
| 17 | 2 | TK 47 | 3 | 18667548 | 18668114 | NA |  |  |
|  |  | TK 38 |  |  |  |  |  |  |
| 18 | 2 | TK 47 | 3 | 43706755 | 43706960 | ABHD5 | 51099 |  |
|  |  | TK 47 |  |  |  |  |  |  |
| 19 | 2 | TK 47 | 3 | 71560835 | 71586175 | FOXP1 | 27086 | TK 38 , TK 47 |
|  |  | TK 47 |  |  |  |  |  |  |
| 20 | 2 | TK 47 | 3 | 172467438 | 172468147 | TNIK | 23043 |  |
|  |  | TK 47 |  |  |  |  |  |  |
| 21 | 3 | TK 47 | 4 | 39862881 | 39882467 | **RHOH** | 399 | TK 38 |
|  |  | TK 47 |  |  |  |  |  |  |
|  |  | TK 47 |  |  |  |  |  |  |
| 22 | 2 | TK 47 | 4 | 39917947 | 39934267 | **RHOH** | 399 | TK 38 |
|  |  | TK 47 |  |  |  |  |  |  |
| 23 | 2 | TK 47 | 4 | NA |  |  |  |  |
|  |  | TK 47 |  |  |  |  |  |  |
| 24 | 2 | TK 47 | 5 | 54392585 | 54403796 | GZMK | 3003 | TK 38 , TK 47 |
|  |  | TK 47 |  |  |  | GZMA | 3001 | TK 38 , TK 47 |
| 25 | 2 | TK 47 | 5 | 66155525 | 66159973 | MAST4 | 375449 | TK 38 |
|  |  | TK 47 |  |  |  |  |  |  |
| 26 | 2 | TK 47 | 5 | 130616970 | 130621874 | CDC42SE2 | 56990 | TK 38 |
|  |  | TK 38 |  |  |  |  |  |  |
| 27 | 2 | TK 38 | 5 | 180082922 | 180082985 | OR2Y1 | 134083 |  |
|  |  | TK 38 |  |  |  |  |  |  |
| 28 | 3 | TK 47 | 6 | 35795213 | 35806517 | FKBP5 | 2289 |  |
|  |  | TK 47 |  |  |  | C6orf81 | 221481 |  |
|  |  | TK 38 |  |  |  | C6orf126 | 389383 |  |
| 29 | 2 | TK 47 | 6 | 90856160 | 90872806 | **BACH2** | 60468 | TK 38 |
|  |  | TK 47 |  |  |  |  |  |  |
| 30 | 2 | TK 47 | 6 | 108245209 | 108247075 | SCML4 | 256380 | TK 38 |
|  |  | TK 47 |  |  |  |  |  |  |
| 31 | 3 | TK 47 | 6 | 117109333 | 117109786 | **KPNA5** | 3841 |  |
|  |  | TK 47 |  |  |  | ZUFSP | 221302 |  |
|  |  | TK 47 |  |  |  |  |  |  |
| 32 | 2 | TK 38 | 6 | 135490514 | 135506571 | **MYB** | 4602 |  |
|  |  | TK 47 |  |  |  |  |  |  |
| 33 | 3 | TK 38 | 7 | 8127857 | 8128163 | ICA1 | 3382 | TK 38 , TK 47 |
|  |  | TK 38 |  |  |  |  |  |  |
|  |  | TK 38 |  |  |  |  |  |  |
| 34 | 2 | TK 47 | 7 | 17144559 | 17145709 | NA |  |  |
|  |  | TK 47 |  |  |  |  |  |  |
| 35 | 2 | TK 38 | 7 | 95938792 | 95939203 | NA |  |  |
|  |  | TK 38 |  |  |  |  |  |  |
| 36 | 6 | TK 47 | 7 | 130314803 | 130346792 | NA |  |  |
|  |  | TK 38 |  |  |  |  |  |  |
|  |  | TK 47 |  |  |  |  |  |  |
|  |  | TK 38 |  |  |  |  |  |  |
|  |  | TK 38 |  |  |  |  |  |  |
|  |  | TK 47 |  |  |  |  |  |  |
| 37 | 2 | TK 47 | 8 | 67056479 | 67056653 | DNAJC5B | 85479 |  |
|  |  | TK 47 |  |  |  |  |  |  |
| 38 | 3 | TK 38 | 8 | 82168914 | 82181372 | **PAG1** | 55824 | TK 38 , TK 47 |
|  |  | TK 38 |  |  |  |  |  |  |
|  |  | TK 47 |  |  |  |  |  |  |
| 39 | 2 | TK 47 | 8 | 101578514 | 101579619 | NA |  |  |
|  |  | TK 47 |  |  |  |  |  |  |
| 40 | 3 | TK 47 | 8 | 121829898 | 121840917 | SNTB1 | 6641 |  |
|  |  | TK 47 |  |  |  |  |  |  |
|  |  | TK 47 |  |  |  |  |  |  |
| 41 | 2 | TK 47 | 8 | 131327000 | 131327804 | DDEF1 | 50807 |  |
|  |  | TK 38 |  |  |  |  |  |  |
| 42 | 2 | TK 38 | 8 | 134147966 | 134156820 | SLA | 6503 | TK 38 , TK 47 |
|  |  | TK 38 |  |  |  | TG | 7038 | TK 38 , TK 47 |
| 43 | 2 | TK 47 | 9 | 5573262 | 5573421 | NA |  |  |
|  |  | TK 47 |  |  |  |  |  |  |
| 44 | 2 | TK 47 | 9 | 19989174 | 19989286 | NA |  |  |
|  |  | TK 47 |  |  |  |  |  |  |
| 45 | 2 | TK 38 | 9 | 126664636 | 126670392 | RPL35 | 11224 |  |
|  |  | TK 47 |  |  |  | ARPC5L | 81873 |  |
|  |  |  |  |  |  | WDR38 | 401551 |  |
| 46 | 2 | TK 47 | 9 | 133572156 | 133594013 | RAPGEF1 | 2889 | TK 38 , TK 47 |
|  |  | TK 47 |  |  |  |  |  |  |
| 47 | 3 | TK 47 | 10 | 6142253 | 6166486 | **IL2RA** | 3559 | TK 38 , TK 47 |
|  |  | TK 47 |  |  |  | RBM17 | 84991 | TK 38 , TK 47 |
|  |  | TK 47 |  |  |  |  |  |  |
| 48 | 2 | TK 38 | 10 | 73748802 | 73748839 | DDIT4 | 54541 | TK 38 |
|  |  | TK 47 |  |  |  | DNAJB12 | 54788 | TK 38 |
| 49 | 2 | TK 47 | 10 | 129750930 | 129750941 | **PTPRE** | 5791 | TK 38 , TK 47 |
|  |  | TK 47 |  |  |  |  |  |  |
| 50 | 2 | TK 47 | 11 | 6367901 | 6369091 | APBB1 | 322 |  |
|  |  | TK 47 |  |  |  | SMPD1 | 6609 |  |
|  |  |  |  |  |  | HPX | 3263 |  |
| 51 | 2 | TK 38 | 11 | 47996615 | 47997625 | PTPRJ | 5795 | TK 38 , TK 47 |
|  |  | TK 47 |  |  |  |  |  |  |
| 52 | 2 | TK 38 | 11 | 60596040 | 60605807 | CD5 | 921 |  |
|  |  | TK 47 |  |  |  |  |  |  |
| 53 | 2 | TK 47 | 11 | 72358075 | 72358079 | FCHSD2 | 9873 |  |
|  |  | TK 47 |  |  |  |  |  |  |
| 54 | 2 | TK 47 | 11 | 85529833 | 85532812 | NA |  |  |
|  |  | TK 47 |  |  |  |  |  |  |
| 55 | 2 | TK 47 | 11 | 101669703 | 101694122 | **BIRC3** | 330 | TK 38 , TK 47 |
|  |  | TK 47 |  |  |  | BIRC2 | 329 | TK 38 , TK 47 |
| 56 | 2 | TK 38 | 11 | 127695521 | 127702206 | NA |  |  |
|  |  | TK 47 |  |  |  |  |  |  |
| 57 | 2 | TK 47 | 12 | 9690490 | 9690584 | KLRB1 | 3820 | TK 38 , TK 47 |
|  |  | TK 47 |  |  |  | CLEC2D | 29121 | TK 38 , TK 47 |
| 58 | 2 | TK 47 | 12 | 12506421 | 12526233 | LOH12CR1 | 118426 |  |
|  |  | TK 47 |  |  |  | DUSP16 | 80824 | TK 38 , TK 47 |
| 59 | 2 | TK 47 | 12 | 12768808 | 12772413 | **CDKN1B** | 1027 |  |
|  |  | TK 47 |  |  |  | GPR19 | 2842 |  |
| 60 | 2 | TK 47 | 12 | 14430559 | 14439399 | **ATF7IP** | 55729 | TK 38 |
|  |  | TK 38 |  |  |  |  |  |  |
| 61 | 2 | TK 38 | 12 | 108802039 | 108811103 | GLTP | 51228 |  |
|  |  | TK 38 |  |  |  | TRPV4 | 59341 |  |
|  |  |  |  |  |  | TCHP | 84260 |  |
| 62 | 2 | TK 47 | 12 | 115048300 | 115067329 | MED13L | 23389 |  |
|  |  | TK 38 |  |  |  |  |  |  |
| 63 | 2 | TK 38 | 13 | 98949412 | 98954060 | TM9SF2 | 9375 | TK 38 , TK 47 |
|  |  | TK 38 |  |  |  |  |  |  |
| 64 | 2 | TK 38 | 14 | 24206514 | 24210437 | GZMB | 3002 | TK 38 , TK 47 |
|  |  | TK 47 |  |  |  |  |  |  |
| 65 | 2 | TK 47 | 14 | 49477778 | 49482979 | **ARF6** | 382 | TK 38 , TK 47 |
|  |  | TK 47 |  |  |  |  |  |  |
| 66 | 3 | TK 47 | 14 | 93494630 | 93512780 | ASB2 | 51676 | TK 38 , TK 47 |
|  |  | TK 47 |  |  |  | FAM181A | 90050 | TK 38 , TK 47 |
|  |  | TK 38 |  |  |  | C14orf48 | 256369 | TK 38 , TK 47 |
|  |  |  |  |  |  | OTUB2 | 78990 | TK 38 , TK 47 |
| 67 | 3 | TK 38 | 15 | 61269825 | 61272680 | RPS27L | 51065 |  |
|  |  | TK 47 |  |  |  | RAB8B | 51762 |  |
|  |  | TK 47 |  |  |  |  |  |  |
| 68 | 2 | TK 47 | 15 | 62962739 | 62972128 | PLEKHO2 | 80301 | TK 38 , TK 47 |
|  |  | TK 38 |  |  |  | ANKDD1A | 348094 | TK 38 , TK 47 |
| 69 | 2 | TK 47 | 15 | 65165654 | 65186500 | **SMAD3** | 4088 | TK 38 , TK 47 |
|  |  | TK 38 |  |  |  |  |  |  |
| 70 | 2 | TK 47 | 15 | 81445578 | 81445746 | HOMER2 | 9455 |  |
|  |  | TK 47 |  |  |  | FAM103A1 | 83640 |  |
|  |  |  |  |  |  | C15orf40 | 123207 |  |
| 71 | 3 | TK 47 | 15 | 84035427 | 84046930 | **AKAP13** | 11214 | TK 38 , TK 47 |
|  |  | TK 47 |  |  |  |  |  |  |
|  |  | TK 47 |  |  |  |  |  |  |
| 72 | 2 | TK 38 | 15 | 89157726 | 89160233 | **BLM** | 641 |  |
|  |  | TK 38 |  |  |  |  |  |  |
| 73 | 2 | TK 38 | 15 | 89201758 | 89216812 | **FES** | 2242 | TK 38 |
|  |  | TK 47 |  |  |  | MAN2A2 | 4122 | TK 38 , TK 47 |
|  |  |  |  |  |  | FURIN | 5045 | TK 38 , TK 47 |
| 74 | 2 | TK 47 | 15 | 91163190 | 91187866 | NA |  |  |
|  |  | TK 47 |  |  |  |  |  |  |
| 75 | 2 | TK 47 | 16 | 20819710 | 20822968 | LYRM1 | 57149 | TK 38 , TK 47 |
|  |  | TK 38 |  |  |  | DCUN1D3 | 123879 | TK 38 , TK 47 |
| 76 | 2 | TK 47 | 16 | 30377284 | 30389106 | ITGAL | 3683 | TK 38 |
|  |  | TK 47 |  |  |  | SEPHS2 | 22928 | TK 38 |
|  |  |  |  |  |  | XTP3TPA | 79077 | TK 38 |
| 77 | 3 | TK 47 | 16 | 55581716 | 55596044 | CETP | 1071 | TK 38 , TK 47 |
|  |  | TK 47 |  |  |  | NLRC5 | 84166 | TK 38 , TK 47 |
|  |  | TK 47 |  |  |  |  |  |  |
| 78 | 2 | TK 47 | 16 | 87902753 | 87909706 | ANKRD11 | 29123 | TK 38 , TK 47 |
|  |  | TK 38 |  |  |  |  |  |  |
| 79 | 2 | TK 38 | 17 | 22820528 | 22820760 | KSR1 | 8844 | TK 38 , TK 47 |
|  |  | TK 38 |  |  |  |  |  |  |
| 80 | 2 | TK 47 | 17 | 26171470 | 26176323 | CRLF3 | 51379 |  |
|  |  | TK 47 |  |  |  | ATAD5 | 79915 |  |
| 81 | 3 | TK 47 | 17 | 27681847 | 27701380 | ZNF207 | 7756 | TK 38 , TK 47 |
|  |  | TK 47 |  |  |  | C17orf75 | 64149 | TK 38 , TK 47 |
|  |  | TK 47 |  |  |  |  |  |  |
| 82 | 3 | TK 47 | 17 | 35148459 | 35148562 | **ERBB2** | 2064 |  |
|  |  | TK 47 |  |  |  | **GRB7** | 2886 | TK 38 |
|  |  | TK 47 |  |  |  | C17orf37 | 84299 | TK 38 |
| 83 | 2 | TK 47 | 17 | 43136967 | 43154096 | TBKBP1 | 9755 | TK 38 , TK 47 |
|  |  | TK 47 |  |  |  | **TBX21** | 30009 | TK 38 , TK 47 |
| 84 | 3 | TK 38 | 17 | 55263659 | 55291288 | TMEM49 | 81671 | TK 38 , TK 47 |
|  |  | TK 38 |  |  |  | RPS6KB1 | 6198 |  |
|  |  | TK 38 |  |  |  | TUBD1 | 51174 |  |
| 85 | 2 | TK 38 | 17 | 61937150 | 61944072 | PRKCA | 5578 | TK 38 |
|  |  | TK 38 |  |  |  |  |  |  |
| 86 | 3 | TK 47 | 17 | 62827735 | 62828080 | PSMD12 | 5718 | TK 38 , TK 47 |
|  |  | TK 47 |  |  |  | PITPNC1 | 26207 | TK 38 , TK 47 |
|  |  | TK 47 |  |  |  |  |  |  |
| 87 | 3 | TK 47 | 18 | 2948458 | 2970218 | LPIN2 | 9663 | TK 38 |
|  |  | TK 47 |  |  |  |  |  |  |
|  |  | TK 47 |  |  |  |  |  |  |
| 88 | 2 | TK 47 | 18 | 9106962 | 9107129 | NDUFV2 | 4729 | TK 38 , TK 47 |
|  |  | TK 47 |  |  |  | ANKRD12 | 23253 | TK 38 , TK 47 |
| 89 | 2 | TK 47 | 18 | 13551086 | 13553044 | C18orf1 | 753 | TK 38 , TK 47 |
|  |  | TK 47 |  |  |  |  |  |  |
| 90 | 2 | TK 47 | 18 | 19801506 | 19803449 | TTC39C | 125488 | TK 38 |
|  |  | TK 38 |  |  |  |  |  |  |
| 91 | 2 | TK 47 | 18 | 44704711 | 44712065 | SMAD7 | 4092 | TK 38 |
|  |  | TK 47 |  |  |  |  |  |  |
| 92 | 2 | TK 47 | 19 | 2003943 | 2004582 | MKNK2 | 2872 | TK 38 , TK 47 |
|  |  | TK 38 |  |  |  | BTBD2 | 55643 |  |
|  |  |  |  |  |  | C19orf36 | 113177 | TK 38 , TK 47 |
|  |  |  |  |  |  | MOBKL2A | 126308 | TK 38 , TK 47 |
| 93 | 2 | TK 38 | 19 | 3012723 | 3012856 | AES | 166 |  |
|  |  | TK 38 |  |  |  | GNA11 | 2767 |  |
|  |  |  |  |  |  | TLE2 | 7089 |  |
| 94 | 2 | TK 38 | 19 | 13135254 | 13143230 | STX10 | 8677 | TK 38 , TK 47 |
|  |  | TK 38 |  |  |  | **IER2** | 9592 | TK 38 , TK 47 |
|  |  |  |  |  |  | TRMT1 | 55621 | TK 38 , TK 47 |
|  |  |  |  |  |  | BTBD14B | 112939 | TK 38 , TK 47 |
| 95 | 2 | TK 47 | 19 | 46752492 | 46752859 | CEACAM21 | 90273 | TK 38 , TK 47 |
|  |  | TK 47 |  |  |  |  |  |  |
| 96 | 2 | TK 47 | 20 | 5047850 | 5048986 | **PCNA** | 5111 |  |
|  |  | TK 38 |  |  |  | CDS2 | 8760 |  |
|  |  |  |  |  |  | C20orf30 | 29058 |  |
| 97 | 2 | TK 47 | 20 | 30578782 | 30579573 | C20orf112 | 140688 |  |
|  |  | TK 47 |  |  |  |  |  |  |
| 98 | 3 | TK 47 | 20 | 33792792 | 33794838 | NFS1 | 9054 | TK 38 |
|  |  | TK 47 |  |  |  | **RBM39** | 9584 | TK 38 |
|  |  | TK 47 |  |  |  | **PHF20** | 51230 | TK 38 |
|  |  |  |  |  |  | C20orf52 | 140823 | TK 38 |
| 99 | 2 | TK 38 | 20 | 46809386 | 46810161 | PREX1 | 57580 | TK 38 , TK 47 |
|  |  | TK 47 |  |  |  |  |  |  |
| 100 | 2 | TK 47 | 21 | 35319895 | 35335039 | **RUNX1** | 861 | TK 38 , TK 47 |
|  |  | TK 47 |  |  |  |  |  |  |
| 101 | 2 | TK 38 | X | 135512746 | 135531355 | CD40LG | 959 | TK 38 |
|  |  | TK 47 |  |  |  |  |  |  |
| 102 | 2 | TK 47 | X | 135663175 | 135690154 | ARHGEF6 | 9459 |  |

The Table show 102 integration clusters identified in post infusion T cell. For each cluster, the table reports the number of hits determining the cluster (cluster dimension), the source (patient), the genomic position (chromosome, position start and end), the target genes (gene symbol and entrez gene) and the genes in common with clusters identified in pre-infusion T cells. Cancer-associated genes (defined in <http://microb230.med.upenn.edu/protocols/cancergenes.html>) are indicated in bold.
